# Advantages of and Barriers to Crafting New Technology in Healthcare Organizations: A Qualitative Study in the COVID-19 Context

**DOI:** 10.3390/ijerph19169951

**Published:** 2022-08-12

**Authors:** Sari Mansour, Sarah Nogues

**Affiliations:** School of Business Administration, TÉLUQ University of Quebec, Montreal, QC H2S 3L5, Canada

**Keywords:** adoption job crafting, adaptive expertise, new technology, well-being, sustainability, workload, virtual teamwork, communication, human resource management

## Abstract

Nursing professionals are constantly required to adapt to technological changes, and especially so in the wake of COVID-19, which has prompted the development of new digital tools. A new and specific form of job crafting in relation to new technology has recently emerged in the literature; that is, adoption job crafting. However, little is known about this specific form of job crafting, especially within the pandemic context. We aim, in this study, to explore the advantages of and barriers to adoption job crafting. We used NVivo software to analyze 42 semi-structured interviews conducted during COVID-19. Our findings revealed that nurses had proactive and positive attitudes toward new technology (adoption job crafting) to enhance efficiency, sustainability, well-being, virtual teamwork, communication, and knowledge sharing. We also identified many barriers to adoption job crafting due to several organizational obstacles, such as the lack of human resource management practices, especially training, and the characteristics of the technology used. We contribute to the literature by documenting innovative cases of and barriers to adoption job crafting, which have not been explored before. These findings stress the necessity to adopt human resources practices, especially training, to foster positive job crafting among nurses and safeguard their adaptive expertise.

## 1. Introduction

In the context of the fourth industrial revolution, marked by technological advancement, including the rise of artificial intelligence (AI), the nursing profession is bound to profoundly change and nurses will have to adapt to new ways of working [1,2]. It is anticipated that new digital technologies will become more widespread and affordable, and some expect them to solve the human resources crisis in the healthcare sector, as well as improve the work environment and conditions for healthcare professionals [3]. It can also be supposed that they may bring solutions to the challenges posed by the pandemic crisis, for instance, with the rapid development and extensive use of digital collaboration tools, such as the online platforms Zoom or Teams. Despite these advantages, new technological tools may cause some challenges in terms of occupational health and safety (OHS). Indeed, the COVID-19 crisis revealed how many frontline and often low-skilled workers (e.g., healthcare and retail business) who delivered products and services to those working from home were more exposed than others to health risks. The ambiguity and uncertainty caused by COVID-19 have compounded these issues, putting employees at high risk of burnout, and seriously threatening and weakening the health systems in some countries [4]. Thus, it could be that robots, new technological tools, and other forms of artificial intelligence will not replace healthcare personnel, including nurses, but they will transform work practices in complex and unforeseen ways (e.g., deskilling, role expansion, and task crafting) [5,6]. Digital transformation has been more present for years in the health sector than in other industries, and is becoming increasingly relevant for researchers and practitioners [7,8,9].

In such a context, the nursing profession is expected to be profoundly transformed and will have to adapt to new ways of working [1,2] to remain relevant and irreplaceable [10]. Regarding the pandemic context, there is a need to investigate the best practices to help employees adjust to their chaotic work environment [11]. Indeed, technological changes can trigger various reactions in employees, ranging from proactive adaptation to resistance to change [12,13]. For example, the use of electronic medical records could lead to negative results if the staff are not consulted during the process of implementing these records [14]. In a recent paper, Holford [15] (p. 1) warned against a potential erosion of nurses’ “adaptive expertise”, a specific and intuitive form of knowledge broadly defined as a “capability to solve problems within new or novel situations”, also referred to elsewhere as “adaptive performance” or “performance adaptation” [16]. This highlights the risk of a growing gap between those who will show adaptive expertise and those who will not. Therefore, it is important that nurses and other healthcare professionals become proactive agents to avoid potentially negative consequences [7,15] and to achieve person-focused care and participation [17].

In the context of change, job crafting (proactive initiatives not imposed by others or by job description) would make it possible to cope with change and to optimize working conditions [18]. For example, recent research highlighted that job crafting behaviors enhanced employees’ adaptive performance and vice versa [16,19]. Regarding the introduction of new technology in particular, scholars have identified the practice of “adoption job crafting”, that is, “the active and goal-directed use of technology and other sources of knowledge to alter the job and enhance a work process”, which includes using new technology to facilitate communication, to enhance work organization, or to automate tasks to reduce the potential for errors [20,21]. Bruning and Campion [22] (p. 509) stated that “it represents a form of resource crafting as it involves adopting, importing, or integrating environmental technology or knowledge-based resources into one’s formal work role”. This form of job crafting “requires higher job autonomy, job complexity, and other job crafting opportunities” and has many benefits, such as better performance, efficiency, teamwork, process improvement, as well as lower work–home conflict and higher cognitive engagement [22]. However, to what extent are nurses proactive and assisted in the implementation of new technology at work? Job crafting behaviors are generally shaped by work context [23], such as HRM practices [24], including job design and training [25,26]. This will require a bottom-up approach to implementing technological change [7] to support employees in adapting to their work environment transformed by the pandemic [11]. Therefore, healthcare organizations must take active action and find sustainable solutions to protect the health and well-being of professionals [27] and to sustain their healthcare system by transforming the fundamental practices of human resource management (HRM) [28].

However, the impacts of these emerging technologies to date are largely uncertain and remain unexamined by the nursing profession. There is a dearth of knowledge regarding the possible consequences of technological advancement on nursing personnel and their overall expertise, including their ability to offer empathic care [2]. Kraus et al. [7] highlighted the scarcity of research in this area and thus invited researchers to investigate these questions further. Similarly, Taiminen et al. [8] concluded that research on the adaptability of the health system to the digital 4.0 revolution is only in its infancy. Vrontis et al. [6] pointed out, in their meta-analysis, that technology would affect the well-being of employees, change their tasks, replace certain jobs, and require new skills. In addition, the recently identified adoption job crafting remains largely unexplored by the literature, and deserves more research attention in the context of rapid technological changes, especially in specific occupations [22]. As much as there is a need to pursue identifying barriers to job crafting among various groups of healthcare professionals [29], it is important to identify barriers to the proactive adoption of new technology amongst nursing professionals [1]. Our research seeks to explore the practice of adoption job crafting in the nursing profession more deeply by identifying its advantages and challenges. More precisely, in a qualitative study conducted during the COVID-19 pandemic in Quebec, Canada, we aimed to provide new insight and a deep investigation into the outcomes of adoption job crafting in terms of adaptive expertise, knowledge sharing, well-being, and other job outcomes. Moreover, we sought to identify barriers to adoption job crafting, such as work intensification, lack of HRM practices, and the characteristics of the new technology, as moderating variables. To our knowledge, this is the first study to explore such a model.

Our research contributes to the advancement of knowledge in the field of new technology and job crafting by focusing on a specific type, that is, adoption job crafting, instead of testing an overall score of job crafting, as was the case in prior research [23]. This allows us to deeply explore this form in the context of technological change, which can advance our understanding of its antecedents and outcomes.

### 1.1. New Technological and Nurses’ Adaptive Expertise

Digital technologies include a wide range of domains: AI, automation technologies (e.g., robotics and drones), assisted living technologies or smart homes technologies, clinical decision support systems, electronic health records, mobile health, telemedicine, personalized healthcare, social media and online information (Internet), and virtual and augmented reality, which all entail potential benefits and pose challenges [30]. These new devices are changing nurses’ relationships with patients; for instance, telemedicine allows monitoring the elderly or individuals with specific diseases (such as cardiovascular or diabetes) from home, instead of having them at the hospital [31]. It is assumed that “nurses will be afforded additional time in their roles with patients to perform human-focused and more complex care activities” which “may allow [them] to hone their skills in other facets of their roles, rather than as collectors of data and tenders of machines and monitors” [2] (p. 8).

While emerging digital technologies (e.g., electronic health records, personalized healthcare, decision-support tools, and mobile devices) have already started transforming the healthcare landscape, the use of AI technologies is presently not so widespread [2]. Recent research suggests they can reduce nurses’ workload [32], allowing them to focus on more essential aspects of nursing, such as empathic and compassionate work with the patient [2] or direct patient care [32]. In this way, new technologies, including AI, seem to require nurses to adapt the way they traditionally perform their job. Observations by Sergeeva et al. [5] following the introduction of the Da Vinci surgical robot already indicate such changes in nurses’ skillsets, leading them to develop new skills and to enlarge their role in performing tasks that would previously be reserved for surgeons. The authors report an extension of nurses’ responsibility and autonomy which generated mixed reactions amongst participants, some enjoying the enhancement of their role and others feeling that they lacked proper training to perform these new tasks [5].

The concept of adaptive expertise or performance has not been defined consistently throughout the literature—some researchers have described it as a behavior aiming “to meet the demands of a new situation or event or a changed environment” [33] (p. 615), while others defined it as a willingness or an ability “to change cognitions and behaviors to adapt to changing environments” [16] (p. 4). Moreover, Holford [15] emphasized a concern that intelligent technologies may decrease professionals’ likeliness to rely on their own intuition to solve problems. Taking the case of airline pilots, he showed how the loss of adaptive expertise amongst these safety professionals has, in some cases, led to dire consequences, which may have been avoided if they had been able to deal with arising situations by tapping into their own intuition without having to rely on a machine. This stresses the need for nursing professionals to exert their adaptive performance and be proactive agents of change to remain relevant [2], and to ensure public health [15]. In this light, what determines nurses’ likeliness to retain their adaptive expertise in the face of technological change? A look into the employee proactivity literature with the concept of “adoption job crafting” may shed light on this question.

### 1.2. Job Crafting and Adaptive Expertise

Employees may adopt job crafting behaviors because of organizational change; that is, they may bring changes to certain aspects of their job to move it closer to their personal preferences or make the job more meaningful [26]. They may bring such changes in the job’s tasks, their relationships at work, or their cognitive work role perceptions. Employees engage in task crafting by “adding extra tasks, altering the scope or nature of tasks, and developing skills and abilities” [21]. Amongst healthcare professionals, “caring moves” were identified as specific job crafting behaviors involving developing and nurturing the relationship with patients [34]. Job crafting is closely linked to employee creativity and innovation, as it has been described as a “process by which individuals initiate and create change over time” [22,26]. Innovation practices have been identified as a crafting strategy, with employees manifesting job-expanding ideas involving an expansion of their responsibilities and abilities [35].

In their meta-analysis, Lichtenthaler and Fischbach [36] emphasized the existence of two distinct forms of job crafting, yielding different employee and organizational outcomes: promotion-focused (or “approach”) and prevention-focused (or “avoidance”) job crafting. Employees who engage in promotion-focused job crafting are focused on making things happen, such as increasing their job resources or challenging job demands, with a proactive attitude. Such proactivity makes the employees more likely to be receptive to organizational changes and to take actions aimed at improving their work environment [37,38,39]. Promotion-focused job crafting has been associated with positive outcomes in terms of employee health, motivation, and performance. Recent studies identified a positive link with adaptive performance, such that approach job crafting enhances employee adaptive performance and vice versa [16,19]. Conversely, employees who engage in prevention-focused or avoidance job crafting adopt an attitude geared towards keeping things from happening. Avoidance job crafting has been identified as a counterproductive work behavior that reduces job performance and increases burnout [36], and is also detrimental to adaptive performance [35]. Job crafting, therefore, seems to be an important avenue to explore when studying the impact of technological changes on nurses’ adaptive expertise.

### 1.3. Human Resource Management Practices and Adoption Job Crafting

Nurses have been reproached with passivity regarding the anticipation of and adaptation to new technology [17], but to what extent does their work environment allow them to be such proactive agents of change? Indeed, poorly managed organizational change may lead to counterproductive behaviors amongst employees. Although job crafting is a bottom-up process, it can only be achieved if employees perceive the necessary conditions to make it effective and more likely to generate positive or negative experiences [21,37]. Job crafting behaviors are generally shaped by work context [17], such as HRM practices [24], including job design and training [25,26]. Therefore, such proactivity and involvement required from nurses imply the presence of bottom-up, as well as top-down, processes, for which a high degree of interaction and communication between nurses and management would be expected. Do nurses have leeway to engage in job crafting behaviors to adapt to technological changes? It is particularly important in the context of COVID-19 to know how nurses can be agents of technological change, as healthcare institutions will need to find new solutions to many challenges arising in various parts of their operations due to COVID-19 [11]. Answering these questions will help shed a light on the impact of technological change on nurses’ adaptive expertise and other job outcomes.

### 1.4. Theoretical Framework and Propositions

Bakker and Demerouti [40], building on resource conservation theory [41], demonstrated on the one hand how employees enter a cycle of losing resources because of work stressors (overload, emotional demands, increased use of technology, lack of time, etc.) leading them to burnout and negative behaviors (intention to quit, poor performance at work, etc.). On the other hand, they pointed out that job crafting activates a cycle of resource gains (e.g., use of skills, etc.), leading to positive outcomes, such as well-being and performance [42]. However, Hobfoll’s theory indicated that organizational resources are often beyond the control of individuals [43]. Nurses would, therefore, need key human resource management (HRM) practices to overcome the challenges of digital transformation and to actively engage in it [28]. For instance, high-performance work practices, such as extensive training, empowerment, and participation in decision making, have been linked to increased promotion-focused job crafting [24].

We can, therefore, expect that new technology may reduce the time and effort spent on routine tasks, and thus may create an opportunity for nurses to craft their job and to develop their skills by focusing on value-added tasks. We also hypothesize that HRM practices and organizational resources would play the role of a catalyst or a passageway allowing nurses to invest resources at work (knowledge, time, and energy) to gain more resources [44,45] and thus strengthen their ability to derive benefits from technology; that is, adoption job crafting. The concept of ‘resource caravan passageways’, which is not well explored [46], refers to organizational “environmental conditions that support, foster, enrich, and protect the resources of individuals, sections or segments of workers, and organizations in total, or that detract, undermine, obstruct, or impoverish people’s or group’s resource reservoirs” [45] (p. 119). According to the technology acceptance model (TAM) [47,48], it also seems that the characteristics of the technology play an important role in its adoption. Indeed, according to these authors, a person accepts the technology when they perceive it as useful and allowing their performance at work to be improved, and when it is easy to use and does not require effort. We thus predict that nurses would invest in adoption job crafting when the technology is perceived as easy to use and useful. In other words, the characteristics of the technology would play a moderating role in the relationship between adoption job crafting and job outcomes.

## 2. Materials and Methods

Our study was based on 42 semi-structured interviews with registered nurses based in the province of Quebec, whom we contacted through first author network listings. An initial series of 8 phone interviews were conducted between 9 March 2020 and 9 April 2020, i.e., during the first COVID-19 pandemic outbreak. Another 34 interviews were conducted between September 2020 and the beginning of January 2021, which coincide with the second COVID-19 wave. The group is predominantly female, with only 3 male participants. The participants occupied various positions, and were mostly nurses and nursing advisors, as well as supervisory positions, such as head nurses (see Table 1).

The present study is part of a global research project investigating several key themes regarding the nursing profession and nurses’ work experiences. We devoted one part to the theme of new technology and created questions inspired by the recent literature about the impact of new technology on the profession. The questions were created to capture participants’ views about the potential for new technology to bring solutions to organizational problems (i.e., heavy workload and staff shortages) and how it may affect their role, and also to assess their actual use of technology in the field. Here are the questions asked:

1. Do you currently use technological tools (for example, telemedicine, mobile health, electronic medical records, Santé Québec files, and new software platforms)?
2. Could these technologies lead to you rethinking or reconfiguring your work, your role, and your tasks? How?
3. Do you think that technological innovations could reduce your work overload?
4. Do you use new technology to facilitate communication or collective work?

Importantly, we also inquired whether and how technology could be useful regarding the pandemic crisis. All participants were interviewed individually by video conference or telephone. The majority were conducted by one of the authors, and a few (5) interviews were conducted by a post-doctoral research assistant involved in the project. Each participant was informed of the conditions under which they agreed to participate by a consent form approved by the ethical committee of our university, which they were asked to read before the interview and return with a signature. All agreed to have the conversation recorded.

**Figure 1:**
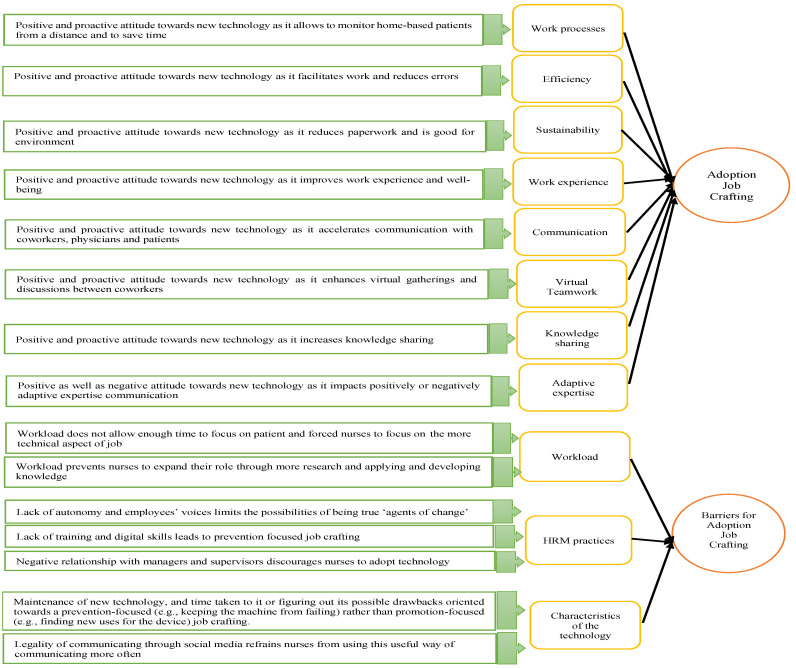
Advantages of and barriers to adoption job crafting.

The mean length of the interviews for both series was 63 min. We immersed ourselves in this recent data through reviewing notes, listening to the interviews, and regular exchanges between the authors. The audio files are currently stored in a safe file-hosting service and in the authors’ locked personal drives. The recordings were transcribed by one of the authors. We opted for qualitative content analysis, which is “a systematic and objective means of describing and quantifying phenomena [49,50]” with the aim “to attain a condensed and broad description of the phenomenon”, and whose expected outcomes are “concepts or categories describing the phenomenon” [51] (p. 108). Hsieh and Shannon [52] indicated that qualitative research can use directed content analysis to explore the prior research findings about a phenomenon that is incomplete more deeply and to validate or extend a theoretical framework. This approach helps to determine the initial codes or relationships between codes by predicting the study’s variables or the relationships between variables [52]. NVivo software v.12 QSR International, Montreal, Canada was used to analyze our data. We followed [53,54] to perform our qualitative analysis. We began our analysis by identifying the topics, codes, and categories that emerged from the interviews. Once this coding step was performed, we continued to the second-order analysis by linking the concepts emerging from our interviews and the existing literature. At this level, we moved between theory, data, and the literature to refine our conclusions, compare them with current theories, and elucidate our contributions [53]. The second-order analysis allowed us to group the emerging concepts into the hierarchical categorizations used in the literature about the adoption of new technology, that is, adoption job crafting behaviors and barriers preventing nurses from the opportunity to adopt and use technology. More precisely, we identified eleven categories arranged into two dimensions. The first dimension was adoption job crafting and included work processes, efficiency, sustainability, work experience, communication, virtual teamwork, knowledge sharing, and adaptive expertise. The second dimension was barriers to adoption job crafting and involved workload, human resource management (HRM) practices (autonomy, employees’ voice, training, digital skills, and relationship with managers), and the characteristics of the new technology. At the end of this analysis phase, these categories were arranged into two dimensions: adoption job crafting and barriers to adoption job crafting. First-order analysis and second-order analysis allowed us to refine our findings and to propose a model of the advantages of and barriers to adoption job crafting (see Figure 1).

## 3. Results

Most participants viewed technological innovations as tools that were likely to improve their experience at work. We observed many cases of positive and proactive attitudes towards new technology, that is, adoption job crafting, and this was observed regarding different technological tools. We also identified many obstacles for adoption job crafting. We firstly present the different cases of adoption job crafting and advantages of this new form of job crafting behavior, and we then explained the different barriers to this behavior.

### 3.1. Advantages of Adoption Job Crafting Behaviors

#### 3.1.1. Adoption Job Crafting Improves Work Processes, Reduces Errors, and Enhances Work Experience

First, some participants described that their workload had been reduced thanks to the introduction of technology, such as the ability to monitor home-based patients from a distance. In the following example, the nurse describes how she has saved a lot of traveling time thanks to the real-time transmission of ventilators at the patients’ homes, which is especially important in rural regions where the areas to cover for traveling nurses are very large:

“*The device did it before, the problem is that I had to move there to go and see. Now I am able to get these measurements remotely, so I would be able to plug into my computer and then go and see*”.

(Oceane, inhalotherapist)

Other nurses also indicated how technology improves her work by reducing time and energy:

“*Just as now, above each bed, there is a pressure cuff that can be programmed, so it will take the pressure regularly. So just go pick it up and see if everything is okay*”.

(Sabrina, Nurse)

This same nurse found that it would improve her work experience and make it more enjoyable:

“*Of course, when they installed pressure monitors for us at the head of each bed, it was more pleasant because we didn’t have to run after a pressure device*”.

Another nurse found that technology such as electronic medical records saves time and energy, and improves well-being:

“*I think we could save time and energy with certain technological tools (….), it did help to save time, it becomes less stressful*”.

(Émili, nurse)

In the following quote, a nurse shares the common practice of using a smartphone app reaching beyond the scope of the tools offered by the hospital to facilitate work and minimize errors by calculating dosages, which allows reducing the potential for errors:

“*Sometimes we have apps on our phone that calculate for us, if we’ve done it before, to see if it works, to make sure it’s reliable*”.

(Mélodie, nurse)

It also seems that technology could reduce paperwork and improve sustainability:

“*I would tell you that the paperwork you have to do a good three, four hours certainly (….). Then it is good for the environment too, if we are able to leave the paper and do our electronic notes… If it would be completely electronic, it would reduce the workload, it would be really fun*”.

(Sabrina, Nurse)

#### 3.1.2. Adoption Job Crafting Increases Communication, Virtual Teamwork, and Knowledge Sharing

The pandemic context has seemingly prompted the expansion of telecommuting and increased the use of new communication tools (i.e., Zoom and Teams) allowing virtual gatherings and discussions between coworkers, which, in some cases, reduced useless commuting times for those who could achieve certain tasks from home. Although in-person contact has been reduced in the workplace due to the COVID-19 prevention measures, these new ways of communicating have, in some cases, allowed new opportunities for relational crafting and the development of side projects which might not have developed without the facilitation of Zoom or Teams. In the following quote, this senior nursing advisor states how she and her team members have discovered new, enjoyable ways to collaborate and develop tools together through such platforms:

“*Depending on the platform, I would say yes. For instance, we were talking about Teams earlier, it is so interesting to be able to work on a tool live. We’re able to see each other, talk to each other, to work in document mode…this is extraordinary.*”

(Jeanne, senior nursing advisor)

We mentioned that the use of text messages between colleagues over the past ten years has accelerated and facilitated coordination among teams. One participant was recently provided a smartphone as a new working tool, a very welcome change that she had been waiting for and provided many benefits by accelerating communication with coworkers and physicians, as well as bettering communication with patients in certain cases. Indeed, the following example shows a form of adoption job crafting, as she found an innovative way to improve communication with certain patients thanks to this new working tool:

“*I have a patient with bulbar amyotrophic lateral sclerosis, well for sure speech gets more and more difficult because of his ALS. But it’s good because we can text him*”.

(Oceane, inhalotherapist)

It is worth noting that this nurse described herself as having a proactive personality and reported having some leeway in making changes in her work due to a permissive relationship with her manager, which was not the case for all participants. The creation of Facebook groups among colleagues is an appropriation of existing technologies by employees to share knowledge and exchange about better ways of working:

“*Sometimes there are exchanges about good practices, about what’s new in our field*”.

(Michelle, nurse)

#### 3.1.3. Adoption Job Crafting Enhances Relationship with Patients and Adaptive Expertise

Platforms acting as interfaces with the patients have also become more important following the pandemic outbreak. This change was perceived as allowing nurses to better achieve patient evaluation, which is a core aspect of their expertise and a central concern reported by many who often regretted not having enough time to dedicate to it. Access to care on the receiving end has obviously improved after the provision of these new platforms:

“*It allows for a better evaluation, when I look at all the mental health telemedicine there has been since the pandemic. It allows us to pursue follow-ups with patients (…) while they’re staying home. So, it really removed accessibility and treatment adherence barriers, plus it’s less expensive*”.

(Megane, nurse)

However, some uses of technology may be detrimental to nurses’ adaptive expertise, from a deontological viewpoint. In some cases, the adoption of these technological devices by nurses also causes problems and adapts their expertise in a way that contributes to growing gaps between nurses and, likewise, gaps in access to care for patients:

“*It’s like the use of Internet or cellphones. I’ve seen…currently, there are nurses who contact patients via Facebook or their cellphone number. The cell phone has become a working tool (…) It creates problems with other nurses who don’t like to get involved with that. I’m not paid to answer my phone 24 h/24 h. I need my private life. It is a demand that is created by individuals who try to give themselves importance and don’t know how to set their limits with patients, and who want some favors. In our deontology, we have to keep our professional distances. Because eventually we cannot favor one patient over another*”.

(Judith, nurse)

Technology thus changes the relationships and communication between nurses and patients, thanks to some job crafting by nurses. However, the adoption of new technology without some level of job crafting may put nurses’ adaptive expertise at risk by increasing their distance from patients.

The following participant suggested that robotics taking charge of recording vital signs, with the ability to monitor them remotely, risks introducing more distance from the patient. This in turn represents a risk for nurses‘ expertise, since there are fewer opportunities for “caring moves”:

“*It would probably be done remotely, I would verify the patient’s pulse, then I would click on my computer: yes, everything is ok, it can take it and then the machine can give it to the patient (…) It would be convenient but then again, we get more distant from our patient!*”.

(Marie Claude, nurse)

In the pandemic context, while it was a common theme amongst our participants that the relational and empathic part of their job as nurses was very important and could not be replaced by technology, the following example shows a shift in the way this participant analyzed and prioritized the need to prevent contagion over the patients’ need for human contact:

“*Yes, there is a lack of human warmth, but at the same time in a period of a pandemic like that, the risk is quite great (…) Because you know I agree that we need human warmth, that we need somebody, but at the same time there is a big risk for the rest of the population. So, at such times I think it’s important that the risks are kept to a minimum*”.

(Melodie, nurse)

However, she thought of an innovative way to overcome this dilemma to keep a form of human connection and empathy, despite the necessity to prevent physical contact:

“*But at the same time, there are studies that prove that if there is no human contact, recovery does not happen the right way. But there might be a way to bring that with… a bit like babies in incubators with hands, so maybe with a stretcher and then the hand near the window, you put your hand through the gain, through a glove in a window, maybe it’s not great but it would be less dangerous for the staff, then with a robot doing other care. To limit as much as possible*”.

(Melodie, nurse)

“*If we get robots to move around and go check on patients instead of nurses…the robot isn’t going to catch COVID-19! Which is why technology can help a lot*”.

(Marie-Claude, nurse)

### 3.2. Barriers to Adoption Job Crafting Behaviors

#### 3.2.1. Due to a Heavy Workload

Some participants expressed the concern that they are forced to operate as machines with no time to think and use their adaptive skills, intuition, and judgment to go deeper in the evaluation and analysis of the patient’s condition. This was a concern raised by many, and there was the idea that, through delegating routine tasks, technology could indeed allow for more time for the nurses to understand each patient’s situation and go further in researching their case. A few participants expressed the desire to expand their role through more research and applying and developing knowledge that could benefit situations and value this aspect of their skillset; however, their heavy workload did not allow such time and forced them to focus on the more technical aspect of their job:

“*(…) if we had the time to be an expert for each patient, well we wouldn’t have this problem of: “I look, then I print out what I’ve read, then hey, doctor, fend for yourself.” Y’know? Because I don’t have time to think anymore, you know? Because I no longer have the time to act, I no longer have the time to investigate the file, to do a complex case search*”.

(Constance, nurse)

“*At some point you lose your judgement, because you work like a robot*”.

(France, coordinating nurse)

Besides expanding their role through research activities, the heavy workload also prevented nurses from engaging in caring moves designed to nurture their relationships with the patients and improve their quality of care. Many regretted that the intensification of work was such that they felt like “machines” and “robots” themselves, and in this way, it was perceived by some that narrow AI could help reduce routine tasks and allow more time for job crafting that would make their role more meaningful, either through spending more time on patient evaluation or spending more time with the patient and the families. However, some participants did not believe that technology could reduce the workload and allow such opportunities, especially those with a more pessimistic position regarding the introduction of new technology. In the following quote, the participant suggested that the number of nurses with expert clinical judgment and adaptive skills cannot be reduced because of the growing complexity of patients’ cases:

“*It is not because we have a lot of technology that we will ultimately reduce the number of staff on the floor, because a patient is not a machine, it’s not just that in the patient’s pathology either. The patient is more and more complex. We need people who think and are able to connect the dots…*”.

(Christelle, nurse)

However, one could argue that such a research task could also be carried out by a supercomputer with advanced diagnosis options. This would, in turn, remove job crafting opportunities for nurses through which they could develop and enrich their knowledge and experience regarding various cases of patients. In the case where AI would increasingly take charge of diagnoses and quickly identify the causes of some patients’ anomalies, nurses may lose their drive to nurture this aspect of their profession, and perhaps lose an essential part of their role in the process, especially as they are not encouraged to do so by a system that keeps on increasing the number of patients to whom nurses must attend. However, there was a sense that the quality of care would be at a loss should they lose this opportunity for contact with patients, as the latter’s ability to interact and connect with another human being is a crucial component of their well-being.

#### 3.2.2. Due to a Lack of Human Resource Management Practices

##### Lack of Autonomy and Employees’ Voices

Concerns were raised regarding the limited possibilities for innovation and autonomy.

Employees’ suggestions are not necessarily considered, and they may have to use tools that already exist, but that are not necessarily adapted to their specific situation. There was a sense that management did not consider the peculiarities of each sector. The highly regulated work environment hardly allows for some job crafting or adaptations, which limits the possibilities for being true ‘agents of change’:

“*If there are things that already exist, it’s okay to use them, but it doesn’t necessarily match what you need. You know, they wouldn’t like us to create new tools but at the same time it is… We have to adapt it to our own needs (…) we won’t have the right to create something that belongs to us ‘there’”*.

(Laurence, chief nurse)

A few participants did have some leeway in changing things and proposing new ideas within their work team. While it was not discouraged, there was no particular emphasis from management on encouraging and empowering employees to adopt such proactive attitudes:

“*You know, I’m the person who will set up, who will do the techniques, who will say: we could do that, we will change that (…)—Does your boss take a positive view of this innovative spirit?—I don’t know… she didn’t tell me. But she didn’t tell me otherwise that it wasn’t right, so I dare believe it’s fine!*”.

(Oceane, inhalotherapist)

##### Lack of Training and Digital Skills

Sometimes, the use of technology to enhance or optimize the work was not because of a lack of skills to do so. For instance, an assistant to an immediate superior would have liked to have optimized her database, but has not done so due to a lack of IT skills. Some also reported a lack of training regarding new technology implemented at work:

“*But I still come back to saying that sometimes it would be nice for us to get some help with training, to help us adapt to these new technologies to use them well*”.

(Sabine, nurse)

The presence of training would alleviate the risks of either resistance to change, loss of adaptive skills, or prevention-focused job crafting:

“*People are lazy. If someone does their job for them… there’s always some people who will find a way to do something else, anything instead of working… going on the Internet, texting…*”.

(Karine, nurse)

“*The danger still remains that there are some nurses who… will rely on the fact that the machine is doing its job, but who won’t necessarily use this time to develop and push that other side of our profession. Because right now, that’s kind of what’s happening. They will concentrate on the technique; hurry quickly and then go do something else (…) There is a risk that employees do not use this the right way.—Could this risk be reduced with training?—Yes, I think so*”.

(Laurence, chief nurse)

While training could prevent the development of avoidance crafting and resistance to change, many participants reported a lack of training opportunities in their work environment. In the following example, the participant describes training where nurses are taught how to use new machines or technology:

“*Well, we have to admit that in hospital centers we are given a lot of training as it pertains to equipment…*”.

(Eric, nursing care advisor)

“*I didn’t really have any training; it was during my days (….) I learned to use the software, but I didn’t have any training as such there*”.

(Sabine, Nurse)

However, he misses other kinds of training that would value and encourage the development of the employee’s judgment and initiative:

“*… But rarely will we get training at a professional level. We need to go and find it by ourselves, because it’s pretty rare that we’ll get any interesting training offered by the employer*”.

(Eric, nursing care advisor)

Training that solely focuses on how to learn a new machine does not encourage job crafting behaviors, as nurses are not necessarily encouraged to use their judgment to adopt new technology, but are rather expected to execute tasks without having a say in the process.

##### Negative Relationship with Managers and Supervisors

Some discouragement could be found regarding the adoption of job crafting behaviors due to rigidity of the work environment and struggles with management. This participant argued that small changes required a considerable amount of energy to be accepted, which discouraged a proactive attitude and innovative input toward the way the work was completed:

“*This is what we do in our job, we are like caught in a whirlwind and we say to ourselves: it can never get better. It’s like we’re discouraged by all of this. It takes a lot of energy for little changes like that. So, we have become somewhat resigned in our job*”.

(Christelle, nurse)

Regarding the pandemic context in particular, a few participants seemed to have innovative ideas and solutions as to how their practice could be adapted regarding the new situation and its consequences on the organization of work and the delivery of care. One participant working as a nurse in obstetrics reported coming up with a solution to the shortage of personnel in the department, which prevented new mothers from being informed and briefed before they returned home with their newborns. Since there were not enough nurses to attend to every patient, this participant proposed to create a series of videos that could be distributed to mothers and, to a certain extent, offset the issue by ensuring a minimum degree of awareness amongst patients. However, this adoption crafting initiative was repeatedly discouraged by management, despite its potential to satisfy nurses by making their work less hectic, and, from the management’s perspective, ensure a minimum standard of services:

“*There are many solutions, nurses are full of ideas, but up there, it gets blocked*”.

(Blandine, nurse)

It was mentioned that the success of technology to alleviate the workload and allow for employees’ innovative and proactive behaviors would also highly depend on the level of team supervision following the implementation and adoption of this new technology. Cases of resistance to change or negative leadership from some employees within the team were reported, which could hinder the successful implementation of new technology and prevent proactive adoption job crafting:

“*So, it’s a big adaptation. It doesn’t look that bad, but for someone who’s used to their routine and then does a lot of things like that… there’s resistance. At this moment you need to see why there is resistance, what is causing the problem, then it is necessary that the worries, the questions are resolved and then they have become aware a little of their technique, in their way of doing things too, but it’s not necessarily a good time for the whole team*”.

(Laurence, chief nurse)

“*You really need to convince them about the necessity of change*”.

(Monique, chief nurse)

“*For sure this will need to be done gradually. There are a lot of people who are reluctant to change*”.

(Marie-Claude, nurse)

In addition, some participants showed skepticism, not regarding the new technology itself, but regarding the odds that it would last as a permanent tool or practice in the organization. Some useful tools were implemented in the past, but were then removed for reasons unknown to the nurses and without much explanation. This triggered an attitude of resistance to change in some participants, whose experience was that changes often were not followed through, and they perceived that the management did not particularly know what they were doing or took random decisions, and reported the frustration of becoming used to a useful tool and changing one’s way of working before finally seeing this new tool or process removed or replaced by another. Such anticipation likely triggers prevention-focused job crafting, or does not encourage promotion-focused job crafting at least, if there is the idea that the new practice or tool will not be permanent:

“*In the beginning it was Zoom, and then wasn’t Zoom anymore, it was Teams. It’s always different from one to the other…and I’m not from your generation, for me it’s complicated. I have to figure out where the microphone is again, how everything works, etc., etc. Ok now I finally got comfortable working with Teams, but don’t come to back to me six months from now with another gimmick which you know will basically be the same thing… eventually I’m still going to need to adapt to it all over again. I think it’s a waste of time*”.

(Adele, nursing care advisor)

Supervision was also deemed important to monitor how employees adapt to changes. Promotion-job crafting will not necessarily occur, as it was perceived that some employees may not be proactive in the context of a reduced workload through the introduction of narrow AI. That is, they may not take the opportunity to spend more time with the patients, develop caring moves, or expand their role through research, but rather take the opportunity to relax, as was already observed by one participant. Therefore, the introduction of technology could facilitate contraction-oriented tasks, aiming to minimize one’s work tasks.

#### 3.2.3. Due to the Characteristics of the Technology

Various forms and degrees of counterproductive behaviors were observed or reported by some participants, whose alleged causes ranged from maintenance problems to lack of communication, supervision, and training. Indeed, concerns were expressed regarding practical aspects and the maintenance of the new technology. Frustration due to difficulties encountered with the technology introduced was anticipated: the time taken to adapt to the new technology or determine its possible drawbacks and failures can represent as much as the time it is supposed to save, limiting the possibilities for technology to alleviate the workload, and even adding problems to deal with. Such technical obstacles orient toward prevention-focused (e.g., keeping the machine from failing), rather than promotion-focused (e.g., finding new uses for the device), job crafting:

“*Because sometimes devices that do not work well can also cause frustration: we rely on that to succeed in doing something and then in the end it doesn’t work (…) So it has repercussions. The fact that someone fails in something and then they go looking for their colleague to help, well the colleague she does not do her job during that time. She’s going to help you know? So, it’s falling behind too. So, it has different repercussions*”.

(Laurence, chief nurse)

Frustration was thus a common theme that encouraged avoidance job crafting behaviors due to its effect of increasing the workload and bringing new problems. One participant with managing duties suggested that the likeliness of new technology to alleviate the workload would highly depend on the level of maintenance required for these devices.

“*Well, most of the time yes, I find it easier but sometimes it’s a little frustrating to see … it’s always all about having to search, get by, the frustration of wasting time with it. Sometimes I have to ask the secretary why it’s not working; can I do such and such a thing… it’s mostly that*”.

(Sabine, nurse)

Social media, such as Messenger, was sometimes used to facilitate communication between coworkers, although it was not endorsed by the hospital and participants had doubts regarding the legality of communicating through this medium, which prevented them from using this useful way of communicating more often. Therefore, ambiguity regarding the use of technology or what system to choose from those installed hindered adoption crafting and was seen as stressful. In addition, many reported that systems were obsolete, and that technology was insufficient at their workplace. One participant indicated that:

“*Technology is not adapted to nurses’ work” and that there is a “lack of IT tools at work” where they “don’t even have Wi-Fi*”.

(Michelle, nurse)

“*It is a bit stressful because, (….), they have to go to two systems… that’s good complicated*”.

(Blandine, nurse)

## 4. Discussion

### 4.1. Theoretical Contributions

Our findings offer important insights regarding nurses’ adoption job crafting opportunities and views about technology within the pandemic context. Our paper contributes to the job crafting literature by providing empirical evidence of adoption crafting [22] in ways that may or may not benefit nurses’ adaptive expertise, thereby contributing to the emerging body of studies exploring the links between job crafting and adaptive performance. We reveal a potential overlap between the two concepts and identify job crafting as a likely mediator between technological change and adaptive expertise. While some of our results are consistent with the findings of Bruning and Camion [22] regarding the positive effects of adoption job crafting on performance, efficiency, teamwork, and process improvement, our study expands their results by revealing more advantages, such as reducing paperwork and enhancing sustainability, making work more meaningful and less stressful, and increasing knowledge sharing and communication among nurses, coworkers, physicians, and patients. Our findings thus document innovative cases of adoption job crafting [20,21], with participants who described themselves as proactive employees [23] and who had some form of empowerment [24], such as the use of mobile phones to facilitate communication with patients through texts messages, thereby allowing them to develop a better ability to communicate and understand certain patients with specific needs. As identified by the recent literature, the COVID-19 situation has led to the fast implementation of digital technological devices, which, in turn, have led to approach job crafting behaviors in some cases, contributing to nurses’ adaptive expertise. Online platforms have led to better patient evaluation and enhanced teamwork for the creation of work tools. These tools also seem to increase knowledge sharing among nurses. Digital technologies allowing enhanced screening, as well as robots (narrow AI) visiting patients, reduce the risks of contagion and have led some nurses to engage in cognitive crafting, further contributing to their adaptive expertise in the sense that they are willing and able to change their cognitions and behaviors in the face of disrupting changes in their work environment [26]. In this way, the proactive adoption of technology has rather enhanced nurses’ expertise, as opposed to threatening it [15].

However, we also revealed a risk that nurses may use technology in ways that lead them to develop work practices against nursing deontology, which may accentuate the gap between nurses who engage in such forms of adoption crafting and others who do not. Whether employees who make their job more enjoyable in ways that are seemingly detrimental to their role as a professional and to the public can qualify as approach crafting is questionable [36], in the sense that, although this is performed to make the work more enjoyable, it does not necessarily have positive effects on performance, public health, or other outcomes. Therefore, our study contributes to the literature by bringing up this issue of adaptive expertise in the job crafting literature. For instance, our results, while they support the link between job crafting and adaptive performance [16,19], invite future studies to investigate the possible mediating role of job crafting between technological change and adaptive expertise. Attention should also be paid to a potential overlap between the two concepts, as both include behaviors to modify one’s work to either meet organizational goals or make the job more meaningful.

Furthermore, we should highlight that it cannot be expected that nurses will successfully adopt new technologies, regardless of their work environment. There are many causes for resistance to change in this profession [55]. Indeed, not all nurses showed approach job crafting behaviors, especially those who had become more cynical over the years. Not all employees are likely to be proactive in adopting technology, and avoidance job crafting should be expected. Moreover, our results suggest that resistance to change, partly due to perceived past management failures regarding the implementation of new technology, may block approach job crafting, preventing nurses from taking part in the technological shift. Since high-performance work practices have been found to encourage job crafting among employees [23], promoting approach job crafting through such initiatives could prevent resistance to change and foster approach, rather than avoidance, job crafting behaviors. Therefore, approach job crafting must be encouraged through organizational conditions and high-performance work practices [36,56]. Extensive training (valuing the development of nurses’ expertise as opposed to solely focusing on them learning new procedures), empowerment, and participation in decision-making [24] appear as three essential management practices that hospitals should implement to foster approach job crafting and prevent resistance to change.

Our finding expands the previous literature, and, in particular, the study of Bruning and Camion [22], by identifying barriers to this specific type of job crafting. Indeed, we found that high workload, as well as a lack of resources, such as human resource management (HRM) practices (autonomy, employees’ voice, training, digital skills, and relationship with managers), prevented nurses from seizing many opportunities for adoption job crafting. Additionally, many participants have reported that practical aspects, maintenance, upgrading, and legal aspects of new technology prevented them from taking many opportunities to take advantage of technology, and, in some cases, even led to resistance to change. This is in line with the Tam model, which predicts that easy-to-use and useful technology would play an essential role in technological change [47]. HRM and the characteristics of technology could, therefore, play a moderating role and act as a resource caravan passageway [57,58], enhancing or detracting nurses’ resource reservoirs. In the same vein, the characteristics of the new technology played another moderating role in adoption job crafting.

### 4.2. Practical Implications

From a practical viewpoint, as called for by recent research, we provide important insights regarding the barriers to job crafting [29], as well as barriers to the proactive adoption of new technology [1] amongst nursing professionals, particularly in the context of COVID-19, which has prompted the development of digital tools in the healthcare sector. Many participants expressed a frustrated desire to develop innovative ideas or expand their knowledge due to organizational obstacles, including ideas to use technology to solve new problems caused by the COVID-19 pandemic. In this way, while some demonstrated behavior with the intent to meet the demands of the new pandemic situation [33] by engaging in job crafting (including adoption crafting using digital technologies to solve problems), organizational obstacles prevented them from doing so. Thus, the preservation of nurses’ adaptive expertise does not only depend on their own will, but can be threatened by contextual factors, such as organizational obstacles, even when they are willing to be proactive agents of change. This provides important insight regarding our question of what determines nurses’ likeliness to keep their adaptive expertise in the face of technological change. While there is a need to support employees to adapt to their altered work environment in the context of pandemics [11], we observe that their attempts to do so are often discouraged, which is in contradiction to a bottom-up approach in the implementation of technological change [7]. Indeed, it was suggested that adoption job crafting requires certain conditions, such as autonomy [20]. Our findings indicate that promotion-focused, adoption job crafting is also highly dependent on the level of supervision and technical maintenance dedicated to the new technology. Ideally, the introduction of new technology should not pose “new problems”. To think about how new technology may improve work processes, nurses should not be spending time trying to sort out its intricacies and solve unexpected problems. This task should be devolved to maintenance agents or supervisors specifically assigned to this task. Moreover, within the COVID-19 context, technology would help with screening, ensuring less physical contact, and providing more safety with technology, but there is still an awareness and consciousness of the need to maintain an empathic rapport with the patient and find solutions to preserve human contact within this context. Therefore, some nurses made special efforts to rethink their role in this context, highlighting instances of promotion-focused cognitive adoption crafting.

Our findings suggest that technology may enhance the development of nurses’ skills through reducing their workload, thereby allowing opportunities for task-expanding job crafting. Technology may help reduce the workload and allow more time for caring moves [34] and skill enlargement. Some nurses felt like their skills were not used to their full potential and felt that their clinical knowledge was underexploited for the sake of repetitive tasks that they were willing, if not wishing, to delegate. A frustrated desire for task enlargement and role enhancement was thus observed, especially for those who described themselves as proactive with a will to evolve in their career (e.g., pursuit of a Bachelor’s degree). Some nurses in our group were proactive and willing to expand their practice through research into more specific cases and use their skills and knowledge in this way; however, were not able to do so because they were forced to focus on more urgent, technical aspects of their job. In this way, narrow AI could enhance nurses’ adaptive expertise [15] by reducing simple tasks to allow more time with the patient and more research time, and help them recenter to central skills that they wished they had the opportunity to develop. At the same time, we can imagine that more sophisticated forms of AI, used for advanced diagnoses, could also discourage nurses from engaging in innovative behaviors if they feel that their clinical judgment is somehow made obsolete. This relates to the risk of nurses losing their adaptive skills. Through researching a patient’s case, nurses can develop a greater ability for empathy with the patient; therefore, there would be a loss in the care relationship.

Moreover, it should be questioned whether supercomputers can replace nurses’ adaptive expertise and judgment. Replacing this role with machines may keep nurses from engaging in job crafting, which, in turn, may be detrimental to their adaptive expertise and may decrease the quality of care. In short, narrow AI could allow more time for nurses to engage in job crafting, but more sophisticated forms of AI may discourage them from engaging in job crafting. Loss of motivation for job-expanding behaviors may result in losing nurses’ adaptive expertise, as they may limit themselves to performing technical, routine duties without resorting to their judgment or intuition as much as they would if they were engaged in promotion job crafting. Moreover, even in the case of technology allowing more time with the patient, our findings suggest that not all of them will automatically take the initiative to spend more time with the patients and engage in task-enhancing job crafting, nor cognitive job crafting by rethinking their role as coordinators, for instance [2].

As a final note, many participants were in favor of the introduction of new technology as long as it would assist them and benefit their work experience. As a matter of fact, many indicated that their ways of working were obsolete (e.g., still working with manual notes), and were waiting for their employer to make the technological shift. Moreover, some participants reported that the pandemic context had slowed down or stopped the development of new projects, including projects related to the development of technological advances. In this context, there was even more skepticism as to how new resources could be allotted to the maintenance and management of new technology when basic organizational and management needs are not met.

### 4.3. Limitations

This study contains some limitations. First, we designed our interview guide before the first pandemic outbreak, which we did not anticipate and which coincided with the beginning of our first series of interviews. In order not to alter the data collection, we did not change the nature of our questions, except by introducing an additional question about whether technology could provide solutions in the COVID-19 context. We may have developed a more adapted set of questions if we had started our research later. In any case, interviews were conducted over a long time span covering two COVID-19 outbreaks, which may have introduced differences complexifying data analysis. In addition, it should be noted that the level of workload, as a barrier to adoption job crafting, could vary depending on the nurse–patient ratio. Unfortunately, we were unable to establish such a ratio for each nurse interviewed in our study, as in Quebec, such ratios are not implemented everywhere. In addition, we think that the COVID-19 crisis has forced hospitals that used such ratios before to not apply them. It would still be interesting to compare our findings based on the nurse–patient ratio in future studies.

## 5. Conclusions

It is without a doubt that new technology and AI will change the role of nurses and their skillset, and push their occupational boundaries [59]. While robots are unlikely to replace healthcare professionals, “those who use AI will probably replace those who don’t” [3]. (p. 1). Research on the adaptability of the healthcare system to the digital 4.0 revolution is only in its infancy, and one of the most important determinants of adaptive capacity is human capital [1]. Therefore, as the nursing profession is undergoing a transformation, it is important that nurses be proactive agents of this change to avoid the potentially negative consequences of such technological development [7]. This suggests a need to involve the professionals in the process [3], as they should be involved in decision-making regarding which aspects can be delegated to the robots and which cannot [2].

## Figures and Tables

**Table 1:** Description of the participants.

Sample’s Characteristics		*n*	%
Gender	Male	3	7.14
	Female	39	92.86
Age	20–30	4	9.5
	31–40	10	40.48
	41–50	17	38.1
	51–60	7	16.16
	61–70	4	9.52
Position	Nurse	35	83.33
	Inhalotherapist	1	2.38
	Nursing Care Advisor	3	7.14
	Head Nurse	3	7.14
Employer	Hospital	34	80.95
	Clinic	4	9.5
	University Hospital Center	3	7.14
	Community Center	1	2.38
Seniority in Profession	1–5 years	1	2.38
	6–10 years	5	11.9
	11–15 years	9	21.42
	16–20 years	9	21.42
	21–25 years	4	9.5
	26–30 years	6	14.28
	31 years and over	5	11.9
	N.A	3	7.14
Seniority in Position	1–5 years	22	52.38
	6–10 years	9	21.42
	11–15 years	5	11.9
	16–20 years	2	4.76
	N.A	4	9.5

N.A= not available.

## Data Availability

The data presented in this study are available on request from the corresponding author. The data are not publicly available due to privacy.
